# A New Crocodylian from the Late Maastrichtian of Spain: Implications for the Initial Radiation of Crocodyloids

**DOI:** 10.1371/journal.pone.0020011

**Published:** 2011-06-08

**Authors:** Eduardo Puértolas, José I. Canudo, Penélope Cruzado-Caballero

**Affiliations:** Grupo Aragosaurus-IUCA, Paleontología, Facultad de Ciencias, Universidad de Zaragoza, Zaragoza, Spain; Raymond M. Alf Museum of Paleontology, United States of America

## Abstract

**Background:**

The earliest crocodylians are known primarily from the Late Cretaceous of North America and Europe. The representatives of Gavialoidea and Alligatoroidea are known in the Late Cretaceous of both continents, yet the biogeographic origins of Crocodyloidea are poorly understood. Up to now, only one representative of this clade has been known from the Late Cretaceous, the basal crocodyloid *Prodiplocynodon* from the Maastrichtian of North America.

**Methodology/Principal Findings:**

The fossil studied is a skull collected from sandstones in the lower part of the Tremp Formation, in Chron C30n, dated at −67.6 to 65.5 Ma (late Maastrichtian), in Arén (Huesca, Spain). It is located in a continuous section that contains the K/P boundary, in which the dinosaur faunas closest to the K/P boundary in Europe have been described, including *Arenysaurus ardevoli* and *Blasisaurus canudoi*. Phylogenetic analysis places the new taxon, *Arenysuchus gascabadiolorum*, at the base of Crocodyloidea.

**Conclusions/Significance:**

The new taxon is the oldest crocodyloid representative in Eurasia. Crocodyloidea had previously only been known from the Palaeogene onwards in this part of Laurasia. Phylogenetically, *Arenysuchus gascabadiolorum* is situated at the base of the first radiation of crocodyloids that occurred in the late Maastrichtian, shedding light on this part of the cladogram. The presence of basal crocodyloids at the end of the Cretaceous both in North America and Europe provides new evidence of the faunal exchange via the Thulean Land Bridge during the Maastrichtian.

## Introduction

Crocodylia includes the clades Alligatoroidea, Crocodyloidea, and Gavialoidea, which incorporate all of the current species of crocodiles, alligators, caimans and gharials. The basal members of Crocodylia may have had a Laurasian origin, coming to dominate the crocodylomorph associations in Europe and North America during the Late Cretaceous. During the Cenozoic, crocodylians colonized other continents, especially in tropical areas, replacing most of the Mesozoic crocodylomorph faunas [1]–[4].

The fossil record of continental crocodylians of the Late Cretaceous in Asia, South America and Africa is primarily composed of non-eusuchian mesoeucrocodylians [1], [4]. However, the fossil assemblage of the Late Cretaceous of North America and Europe is different. Basal members of Crocodylia have been described from both of these continents, including basal forms such as *Borealosuchus*, alligatoroids such as *Brachychampsa*, *Stangerochampsa*, *Leidyosuchus*, *Deinosuchus* and *Musturzabalsuchus*, and crocodyloids such as *Prodiplocynodon* [1], [4], [5]. This joint occurrence of crocodylians in North America and Europe suggests that the common ancestor for the clade evolved on one of these two continents [1]. In recent years the record of crocodylians from the end of the Cretaceous has increased greatly in southern Europe, including occurrences in Portugal, Spain, Italy and France [1], [4], [6]–[16]. The most common taxa are *Musturzabalsuchus*, *Allodaposuchus*, *Acynodon* and *Massaliasuchus*, although there have also been reports of gavialoids such as *Thoracosaurus* in the south of France [17]. Until now, no representatives of Crocodyloidea had been cited in the Late Cretaceous of Europe. The crocodyloid *Prodiplocynodon langi* Mook 1941 from the Maastrichtian of the Lance Formation in Wyoming (USA) is the only one known so far from the end of the Cretaceous. The Palaeogene saw the diversification of Crocodyloidea and their dispersion throughout the other continents, examples including *“Crocodylus” affinis* Marsh 1871, *Brachyuranochampsa* and *“Crocodylus” acer* Cope 1882 in North America, *Asiatosuchus* in Eurasia, *Kentisuchus spenceri* Buckland 1836 in Europe, and *“Crocodylus” megarhinus* Andrews 1905 in Africa.

In recent years, a great effort has been made to reconstruct the vertebrate succession of the Pyrenees at the end of the Cretaceous. The vertebrate record close to the K/P boundary is very scarce worldwide, and the Pyrenees is one of the few places with fossiliferous sediments from this time interval [18]. The sites of the inland area of western North America are well studied ([19], [20] and the bibliographies therein), and recently efforts have focused on the dinosaur successions of the terminal Cretaceous in Asia [21]. In the light of these considerations, a study of the vertebrates of the Late Cretaceous of the Pyrenees is of great importance in order to provide a more global vision of the history of vertebrates and how it is related to the K/P boundary event. In the Tremp Basin, dinosaur bones and teeth (from hadrosaurs, sauropods and theropods) are abundant, in addition to a large number of dinosaurian ootaxa, nests and tracks [18], [22]–[27].

The Aragosaurus-IUCA group of the University of Zaragoza has been researching the sediments of the Maastrichtian of the Central Pyrenees (Huesca) for 15 years, making it possible to recover a broad collection of vertebrates [24], [28], especially hadrosaurids [27], [29], [30]. In this part of the Pyrenees, primarily fragmentary remains and isolated teeth from various types of eusuchian crocodylomorphs had been identified previously [24]. During the 2008 campaign, an almost complete cranium of a new taxon was recovered from the Elías site (Arén, Huesca). This paper describes the fossil, ascertains its phylogenetic position, and discusses the palaeobiogeographical implications of its occurrence on the Iberian Peninsula.

## Results

### Geographical and Geological Context

**Figure 1:**
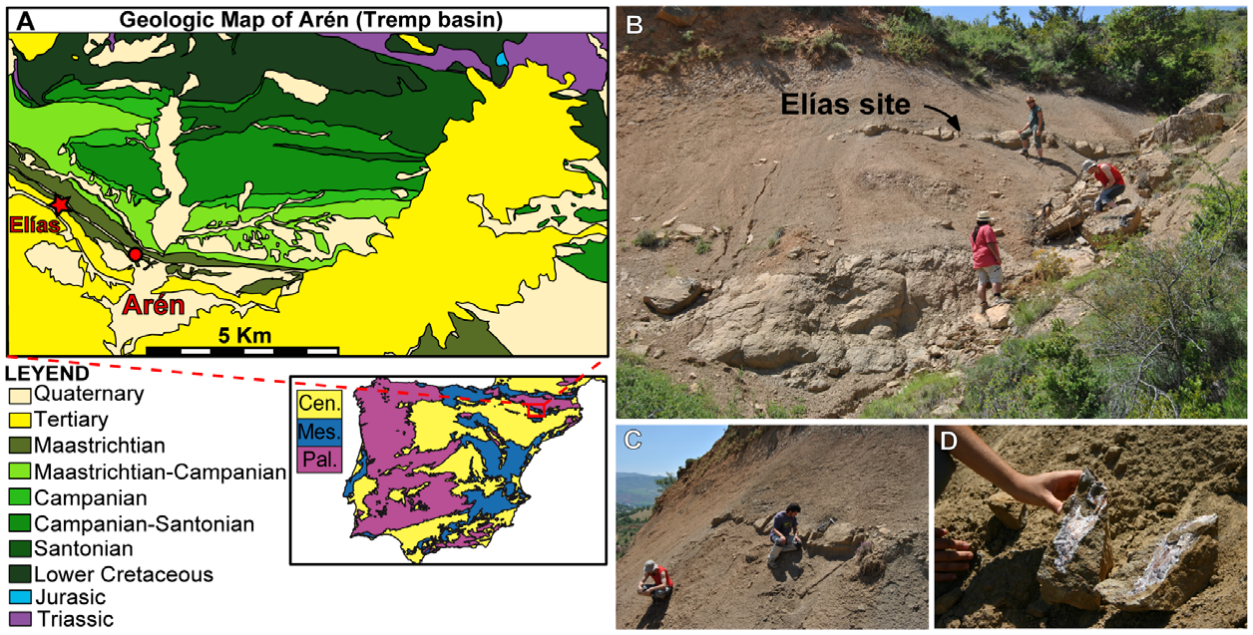
Location where *Arenysuchus gascabadiolorum* (ELI-1) was found. A, Geological map of the Arén locality (the Elías site is indicated by star). B, the Elías site in general view. C, sandstone layer and concrete place where the fossil was collected. D, ELI-1 skull when it was collected.

**Figure 2:**
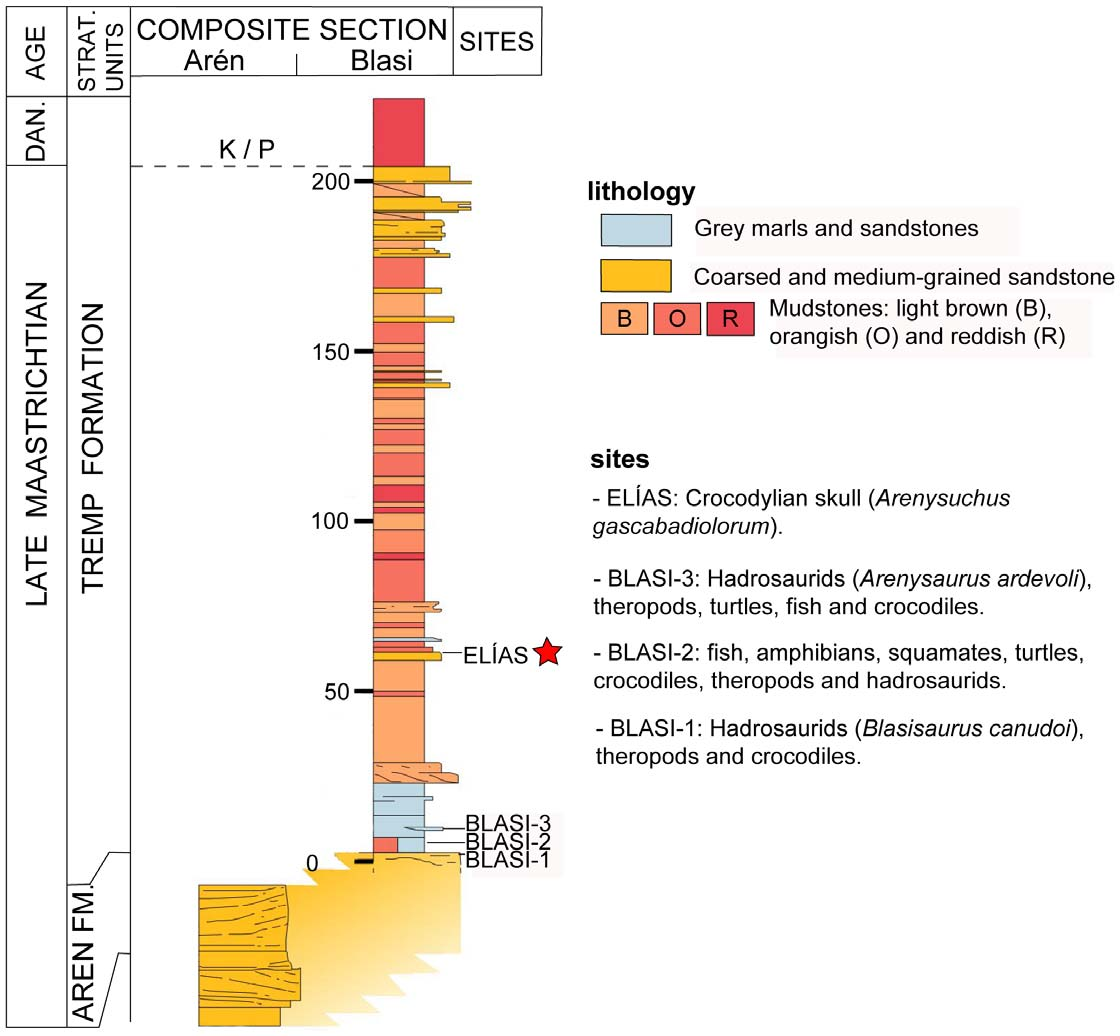
Blasi (left) and Arén (right) stratigraphic sections (Huesca). With the location of the Blasi 1–3 and Elías sites. Modified from Pereda-Suberbiola *et al.* [27].

ELI-1 was recovered from the Elías site, located to the west of Arén (northeastern Huesca, Spain), near Blasi Hill (Fig. 1A). The site is situated on the northern flank of the east-west trending Tremp Syncline. Stratigraphically, the site is situated in the lower red unit (Unit 2) of the Tremp Formation (Figs. 1B, 2), equivalent to the Conques Formation [31]–[33]. The sites Blasi 1–3, from which the lambeosaurine dinosaurs *Arenysaurus* and *Blasisaurus* have recently been described [24], [27], [29], [30], are lower in the same section (Fig. 2).

The continental facies of the Tremp Formation are reddish in colour and have a depth of up to 900 m in the South Pyrenean Central Unit. In its lower part, the Tremp Formation is superimposed upon and interdigitates laterally with mixed-platform marine deposits that are late Campanian-Maastrichtian in age [34]. These deposits are more siliciclastic in the northern outcrops (Arén Sandstone [35]) and more calcarenitic in the southern ones (Les Serres Limestones [36]). Marine sediments of the alveoline limestone Cadí Formation, or marly deposits laterally equivalent to the Figols Group, Ilerdian in age (Lower Eocene, [37]), are above the Tremp Formation. Blasi 1 is located in the upper part of the deltaic facies that form the Arén Sandstone (Arén Formation), whereas Blasi 3 is located in the lower part of the superposed Tremp Formation (Fig. 2). Lithologically, the Elías site comprises very coarse-grained ochre sandstones of polymictic composition and carbonate cement. The fossil-bearing layer has little lateral continuity and pinches out to the east. The depth varies laterally between a few decimetres and one metre (Fig. 1B,C). These sandstones are intercalated with the variegated clays of Unit 2 of the Tremp Formation. Only the cranium discussed in this paper has been found at the Elías site (Fig. 1D); no other vertebrate fossils have been recovered.

The Tremp Formation has been dated by means of guide levels (limits to deposit sequences and rudist horizons at the base of the formation), planktonic foraminifers, and magnetostratigraphy. Guide levels have allowed the high-resolution correlation of the Arén Formation and the lower part of the Tremp Formation with underlying and laterally equivalent marine deposits containing planktonic foraminifers of the *Abathomphalus mayaroensis* Biozone, whose age range is between 68.4 and 65.5 Ma [24], [38]. These data have made it possible to date Unit 2 to between the upper Campanian-lower Maastrichtian and the lower Danian [18], [24], [34]. Within Unit 2, these correlations place the Blasi 1–3 sites in the lower part of this biozone (ca. 68 Ma), and the Elías site, which can be correlated with the Blasi 4–5 sites, in the middle part (ca. 67 Ma). Magnetostratigraphic studies on the same section of the palaeontological sites of Blasi and Elías have identified polarity chrons correlative with Chron C30n (GPTS dated to 67.6 to 65.5 Ma) [27], [33]. As such, the Elías site is late Maastrichtian in age.

### Nomenclatural Acts

The electronic version of this document does not represent a published work according to the International Code of Zoological Nomenclature (ICZN), and hence the nomenclatural acts contained in the electronic version are not available under that Code from the electronic edition. Therefore, a separate edition of this document was produced by a method that assures numerous identical and durable copies, and those copies were simultaneously obtainable (from the publication date noted on the first page of this article) for the purpose of providing a public and permanent scientific record, in accordance with Article 8.1 of the Code. The separate print-only edition is available on request from PLoS by sending a request to PLoS ONE, Public Library of Science, 1160 Battery Street, Suite 100, San Francisco, CA 94111, USA along with a check for $10 (to cover printing and postage) payable to “Public Library of Science”.

In addition, this published work and the nomenclatural acts it contains have been registered in ZooBank , the proposed online registration system for the ICZN. The ZooBank LSIDs (Life Science Identifiers) can be resolved and the associated information viewed through any standard web browser by appending the LSID to the prefix “http://zoobank.org/”. The LSID for this publication is: urn:lsid:zoobank.org:pub:64D3210B-B2D2-4DD3-9100-1611BE66CA7D.

### Systematic Palaeontology

Eusuchia Huxley 1875 [39]

Crocodylia Gmelin 1789 [40], *sensu* Benton and Clark 1988 [41]

Crocodyloidea Fitzinger 1826 [42]

*Arenysuchus* gen. nov.

urn:lsid:zoobank.org:act:EBA4AED1-684C-40E0-BF99-F9FCB16899D9

#### Etymology

*Areny* is named after Arén (Areny in the Catalan language), the locality where ELI-1 was found, and *souchus*, Greek for crocodile, leading to Latin, *suchus*.

#### Type species

*Arenysuchus gascabadiolorum*.

#### Diagnosis

*Arenysuchus* is characterized by the following autapomorphies: infratemporal bar tabular and vertically oriented, with little dorsoventral thickness and extreme lateromedial compression; the dorsal portion of the anterior process of the frontal has a very elongated (≈60 percent of the total rostrocaudal length of the frontal) and lanceolate morphology; the anterior process of the frontal projects strongly beyond the main body of the frontal and extends between the nasals, ending in a sharp point beyond the anterior margin of the orbits and the prefrontal, at the height of the anterior end of the lacrimal. The lacrimal is very wide dorsally, only twice as long as wide (taking its maximum length and maximum width).

#### Differential diagnosis

*Arenysuchus* can be differentiated from other crocodylians on the basis of the following unique combination of characters: *Arenysuchus* presents elevated dorsal rims of the orbits, like the derived crocodyloids, which distinguishes it from the rest of the basal crocodyloids to which it is closely related, such as *Prodiplocynodon*, *Asiatosuchus*, *“Crocodylus” affinis*, *Brachyuranochampsa eversolei* Zangerl 1944 and *“Crocodylus” acer*. The frontoparietal suture of *Arenysuchus* enters the supratemporal fenestra, preserving the plesiomorphic state for Crocodylia. The palatine process of *Arenysuchus* does not extend beyond the rostral end of the suborbital fenestra, as is also the case in other basal crocodyloids such as *Prodiplocynodon langi*, *Asiatosuchus germanicus* Berg 1966, “*Crocodylus” affinis* or “*Crocodylus” acer*, which present the same character. *Arenysuchus* has an occlusion pit between the seventh and eighth maxillary teeth, and all other dentary teeth occlude lingually, which distinguishes it from most crocodyloids, with some exceptions such as *“Crocodylus” affinis*, which presents the same character.

#### Distribution

Late Maastrichtian, North Spain.

*Arenysuchus gascabadiolorum* sp. nov.

urn:lsid:zoobank.org:act:77F72F35-6DA9-47C5-9A3D-96FC7B290A20

#### Holotype

**Figure 3:**
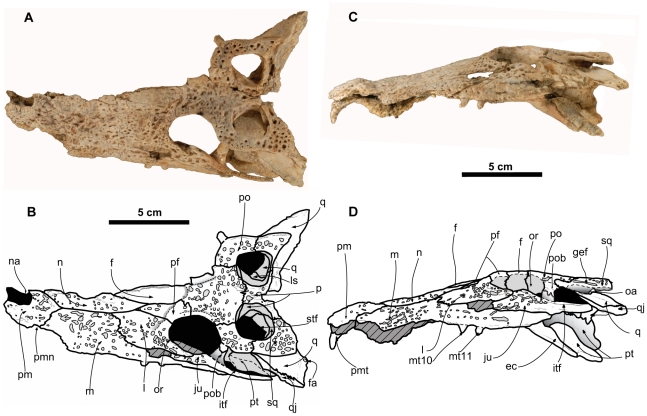
Skull of *Arenysuchus gascabadiolorum* (ELI-1). A–B, dorsal view. C–D, lateral view. Hatched grey pattern represents broken surfaces. Anatomical Abbreviations: ec, ectopterygoid; f, frontal; gef, groove for ear flap; itf, infratemporal fenestra; ju, jugal; l, lacrimal; ls, laterosphenoid; m, maxilla; mt10, maxillary tooth 10; mt11, maxillary tooth 11; n, nasal; na, naris; oa, otic aperture; or, orbit; p, parietal; pf, prefrontal; pm, premaxilla; pmn, premaxillomaxillary notch; pmt, premaxillary tooth; po, postorbital; pob, postorbital bar; pt, pterygoid; q, quadrate; qj, quadratojugal; sq, squamosal; stf, supratemporal fenestra.

**Figure 4:**
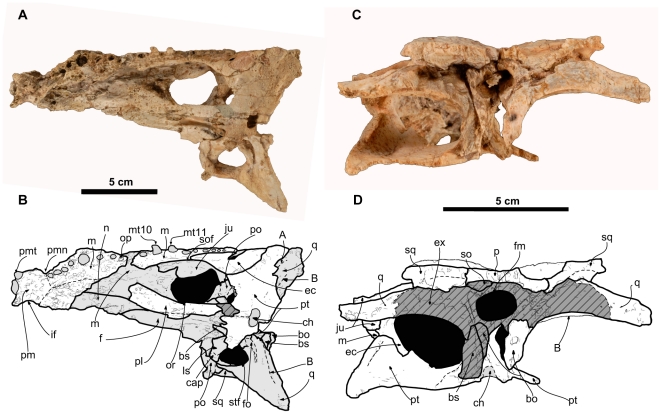
Skull of *Arenysuchus gascabadiolorum* (ELI-1). A–B, ventral view. C–D, posterior view. Hatched grey pattern represents broken surfaces. Anatomical Abbreviations: bo, basioccipital; bs, basisphenoid; cap, capitate process; ch, choana; ec, ectopterygoid; ex, exoccipital; f, frontal; fa, foramen aërum; fm, foramen magnum; fo, foramen ovale; if, incisive foramen; ju, jugal; ls, laterosphenoid; m, maxilla; mt10, maxillary tooth 10; mt11, maxillary tooth 11; n, nasal; op, 7^th^–8^th^ occlusion pit; or, orbit; p, parietal; pl, palatine; pm, premaxilla; pmn, premaxillomaxillary notch; pmt, premaxillary tooth; po, postorbital; pt, pterygoid; q, quadrate; so, supraoccipital; sof, suborbital fenestra; sq, squamosal; stf, supratemporal fenestra; A and B, muscle scars on the quadrate.

**MPZ** ELI-1, partial skull (Figs. 3, 4). The specimen is housed in the Museo Paleontológico de la Universidad de Zaragoza (MPZ), Zaragoza, Aragón, Spain.

#### Referred material

Four teeth from Blasi 2 (MPZ2010/948, MPZ2010/949, MPZ2010/950, MPZ2010/951) (Appendix S3). This material is housed in the Museo Paleontológico de la Universidad de Zaragoza (MPZ), Zaragoza, Aragón, Spain.

#### Etymology

The specific epithet of “*gascabadiolorum*” is dedicated to the researchers José Manuel Gasca and Ainara Badiola, who discovered the holotype.

#### Locality and Age

ELI-1 was gathered from a level of sandstones at the Elías site in Unit 2 of the Tremp Formation (late Maastrichtian) (Fig. 2), approximately equivalent to the Conques Formation. The Blasi sites are situated in the low part of the Tremp Formation (late Maastrichtian). These sites are located in Arén (Huesca), North Spain (Fig. 1A).

#### Distribution

As for genus.

#### Description

ELI-1 is a fairly complete and well-preserved cranium (Figs. 3, 4, measurements in Appendix S5). The mandible, the right half of the maxillary rostrum, most of the teeth, a large part of the palate, and the posterior part of the braincase are absent or incomplete. No associated postcranial material has been found. Internally, the cranium presents small circular depressions produced by the pressure of the detritic clasts of the rock.

The dorsal surface of the cranium displays well-developed ornamentation, comprising pits and grooves. These grooves are more abundant and deeper on the anterior margin of the orbits and on the posterior end of the squamosals. The orbits are large, slightly elongated anteroposteriorly, and display an ovoidal morphology similar to other plesiomorphic crocodylians such as *Prodiplocynodon* and *Leidyosuchus canadensis* Lambe 1907 [43]. The anterior and medial rim of the orbits is upturned. There is an arc-shaped ridge offset by a groove or “spectacle” between the anterior axes of the orbits that is very similar to the one presented by eusuchians such as *Allodaposuchus precedens* Nopcsa 1928 [16], many alligatoroids such as *Leidyosuchus canadensis* [43], and several basal crocodyloids such as *Prodiplocynodon*. The suborbital fenestrae are large and elongated, with straight lateral and medial margins. The supratemporal fenestrae are smaller than the orbits and subrounded. These fenestrae present almost the same lateromedial as anteroposterior width, unlike in other basal crocodyloids such as *Prodiplocynodon* and *Asiatosuchus*, where the openings are somewhat narrower anteroposteriorly. The infratemporal fenestrae are triangular and have a somewhat greater anteroposterior width than the supratemporal fenestrae, very similar to that of the orbits, though their lateromedial width is much less than either.

The premaxillae present an undivided naris. The naris occupies roughly 30 percent of the area of maximum premaxillary width. The naris is longer than it is wide, with the posterior part slightly wider than the anterior part and projecting dorsally. The naris is almost completely enclosed by the premaxillae, and the naris may contact to the nasal, although the right half of the maxillary rostrum is missing and the anterior extent of the nasal cannot be determined with certainty. The narial margin is somewhat inflated, rather than depressed. The dorsal posterior process of the premaxilla is short and wide, and extends to a point level with the third maxillary alveolus, as in nearly all non-longirostrine crocodylians and close relatives with a few exceptions (*Brachyuranochampsa*, some globidontans) [44]. The palatal portion of the premaxillae is not well preserved, but presents a small subcircular incisive foramen close to the first premaxillary alveoli. There is a notch between the premaxillomaxillary suture (Fig. 3A) for the reception of the third and/or fourth tooth of the dentary. At least two alveoli are preserved in the premaxilla, the bigger of which contains a premaxillary tooth, although two or three more alveoli may also have occurred. The preserved premaxillary tooth is conical, and its apex is slightly curved in the distolingual direction. The tooth has a circular cross-section, and a series of very fine basiapically aligned striations ornament the surface. No crests or carinae are preserved, probably due to the poor state of preservation of the surface. We found isolated teeth of similar morphology and with mesial and distal carinae at Blasi 2 [24] (Appendix S3, A).

The maxilla is slightly arched in lateral view, presenting its maximum concavity at the height of the eighth maxillary alveolus. Towards the anterior part, the maxilla has a convex profile with its maximum thickness at the height of the fifth maxillary alveolus; this area also has the maximum width in dorsal view. Posterior to the sixth maxillary alveolus the left and right margins of the maxilla are more parallel to each other in dorsal view. The dorsal surface of the maxilla has a slight circular protuberance posterodorsal to the fifth alveolus. In palatal view, between the tooth row and the suborbital fenestra, the maxilla presents an area of width very similar to the tooth row. This area tends to be much wider in alligatoroids, with the exception of *Diplocynodon styriacus*, where this area is narrower [45]–[48]. The maxilla has at least 15 alveoli, the eleventh and twelfth of which still include the tooth. Given that the part corresponding to the eighth maxillary tooth is broken, and the posterior end of the tooth row is not very well preserved, we infer a total of 16 or 17 maxillary alveoli. The biggest maxillary alveolus is the fifth. There seem to be no depressed areas in the maxilla corresponding to occlusion grooves for accommodating the teeth of the lower mandible, indicating an overbite occlusal pattern, with the exception of the lateral notch between the premaxilla and the maxilla and an occlusion pit between the seventh and eighth maxillary teeth. The preserved maxillary teeth are low, small in size, slightly flattened labiolingually, wider mesiodistally, and with smooth enamel. The eleventh tooth narrows slightly at its base, giving it a somewhat lanceolate morphology. Isolated teeth of similar morphology have also been found at Blasi 2 [24] (Appendix S3, B).

The left nasal is mediolaterally narrow, with an elongated morphology (10 times longer rostrocaudally than it is wide), with a contour similar to that in *Bernissartia fagesii* Dollo 1883 and *Allodaposuchus precedens* [16], [49]. The anterior end of the nasal is not well preserved, so it is difficult to determine the participation in the naris (Fig. 3A), although the nasal is dorsally constricted between the premaxillae and it is possible that the nasal could reach the naris. Posteriorly, the nasals widen abruptly as far as the vertex of the posterior process of the premaxilla, from which point they continue to widen slightly until they reach their maximum width at the point of contact with the lacrimals. From a point level with the eighth maxillary tooth, the nasals are split by the anterior process of the frontal.

The anterior ramus of the jugal is not preserved in the preorbital region, although the bone's morphology can be discerned given that the maxilla and the lacrimal are almost complete in this area. From the anterior ramus, the jugal widens posteriorly, reaching its maximum dorsoventral width at the lateral orbital margin, at which point the posterior ramus becomes narrower again until it reaches the quadratojugal. The jugal forms most of the infratemporal bar, although it is impossible to tell whether the posterior angle of the infratemporal fenestra is formed by the jugal or the quadratojugal, because this region has not been preserved. The jugal forms a large part of the postorbital bar, with an ascending process that starts from the medial surface of the jugal and rises posteromedially until it contacts the ventral part of the postorbital. Two small medial foramina pierce the area anterior and posterior to the postorbital bar. The jugal is raised above the base of the postorbital bar, forming an elevated margin. The shape of the infratemporal bar is very tabular, elongated and lateromedially narrow, similar to *Allodaposuchus precedens*. In lateral view, the infratemporal bar has very little dorsoventral thickness (average thickness of 6 mm) (Fig. 3B), and in dorsal view it presents an extreme lateromedial compression (average width of 3 mm) (Fig. 3A).

The lacrimal is very wide dorsally, lateromedially and anteroposteriorly wider than the prefrontal. The posterior margin of the lacrimal in contact with the orbit is upturned with respect to the dorsal surface of the rostrum and has marked ornamentation on its dorsal surface. The anterior contact between the lacrimal and the maxilla is very sinusoidal, forming two undulations, and the contact with the nasal is broad. A small, dorsoventrally flattened foramen is visible in the anterior wall of the orbital portion of the lacrimal. This may correspond to the lacrimal duct.

The prefrontal has a subtriangular anterior process, with its apex situated between the lacrimal and the nasal. The prefrontals are separated by the frontal and the nasals and do not meet along the midline. The posterolateral end of the prefrontal participates in the anteromedial margin of the orbit and is upturned, like the lacrimal. On its posterior margin, the prefrontal expands medially, causing a narrowing at the beginning of the anterior process of the frontal and giving the frontal a lanceolate shape. The ventral surface of the prefrontal contributes to the anterior portion of the orbit. The entire dorsal surface of the prefrontal is ornamented with subcircular grooves. The prefrontal pillar is longitudinally expanded dorsally, but is more columnar ventrally, as in most crocodylians with the exception of gavialoids. Dorsally, the prefrontal has a fine lateral lamina on the orbital margin beneath the lacrimal and the prefrontal, forming the concavity in which the olfactory bulbs are housed [12] (Fig. 4). The medial process of the prefrontal pillar is not preserved.

The anterior process of the frontal has a smooth, flat dorsal surface, without ornamentation, that is elongated (≈60 percent of the total rostrocaudal length of the frontal), narrow (nine times longer rostrocaudally than it is wide) and lanceolate, showing the plesiomorphic state for Crocodylia. This process is somewhat similar to but longer than that in other eusuchians such as *Allodaposuchus* [12], [16] and *Crocodylus niloticus*. This anterior process is separated from the main body of the frontal and terminates in a sharp point beyond the anterior margin of the orbits and the prefrontal, at the height of the anterior end of the lacrimal (Fig. 3A). The suture with the prefrontals and nasals is smooth, without serrations, and the suture with the prefrontal forms a right angle. The margin of the prefrontal and the frontal between the orbits is elevated in relation to the rostrum, as in derived crocodylids such as *Crocodylus* or *Tomistoma*. The dorsal surface of the frontal between the orbits is markedly ornamented and is practically flat, even though the margin of the orbits is slightly elevated. Posteriorly, the frontal narrows between the supratemporal fenestrae and contacts the parietal at the start of the frontoparietal bar, forming a slightly convex suture. The frontoparietal suture enters the supratemporal fenestra, preventing broad contact between the postorbital and the parietal, as in basal gavialoids and alligatoroids, certain pristichampsines and *Borealosuchus sternbergii* Gilmore 1910, which is plesiomorphic for Crocodylia.

The postorbital is crescentic in dorsal view and forms the dorsal part of the postorbital bar. The postorbital extends somewhat more dorsolaterally than ventromedially on the postorbital bar and is superimposed upon the ascending process of the jugal, the two processes coinciding at roughly the halfway point of the postorbital bar. As occurs in many crocodylians, a slight dorsal swelling on the postorbital bar occurs in the area of contact between the jugal and the postorbital. There is no descending process of the postorbital, a character present in many alligatorids [44], [50], although this area is not very well preserved. As in most basal crocodylians, the postorbital contacts the quadrate medially in the dorsal region of the infratemporal fenestra (Fig. 4A); the postorbital may also contact the quadratojugal, although this area is incomplete and difficult to interpret.

The squamosal constitutes almost a quarter of the boundary of the supratemporal fenestra, corresponding to the posterolateral margin. The squamosal has a fairly horizontal dorsal surface, with ornamentation consisting of deep subcircular grooves, particularly at the posterior end. At this end the squamosal has a horn-shaped posterior projection, although in ELI-1 it is impossible to establish its extent due to incomplete preservation. The squamosal has an elongated and lobular anterior process which extends ventrally to the posterodorsal end of the postorbital (Fig. 3B). The squamosal forms the upper part of the otic opening, and in lateral view it presents the groove for the external ear valve musculature, the rims of which are parallel. This groove traverses the squamosal from the posterior region to roughly the point of contact with the postorbital. The area of contact between the squamosal, the exoccipital, and the posterior region of the quadrate is not well preserved.

The parietal is completely fused, and its most posterior end is missing. The anterolateral margin of the parietal is depressed at the edge of the supratemporal fenestra. The frontoparietal suture enters the supratemporal fenestra (Fig. 3A), and the parietal becomes narrower anteriorly along the anteromedial wall of the supratemporal fenestra, until a point where the parietal wedges out between the frontal and the laterosphenoid, ending in contact with the suture of the postorbital and the frontal. The parietal and the squamosal contact in the most dorsal part of the posterior wall of the supratemporal fossa, but an ascending process from the quadrate prevents contact further ventrally. In dorsal view, the parietal expands very slightly laterally in the area anterior to and between the supratemporal fenestrae, forming the anterior sector of the frontoparietal bar.

The quadratojugal is incomplete, and consequently the development of the quadratojugal spine and the degree of quadratojugal participation in the infratemporal bar cannot be determined. The extension of the quadratojugal to the superior angle of the infratemporal fenestra prevents the quadrate from participating in this fenestra. The preserved part lacks ornamentation.

The palatines are elongate (12–13 times longer rostrocaudally than they are wide), straight and narrow. They are slightly wider at their anterior ends than at their posterior ends, and they expand laterally at the height of the posterior margin of the suborbital fenestra. The anterior end of the palatine and a large part of the maxilla in ventral view have not been well preserved, so the contact between these elements is difficult to resolve. In spite of this, it seems that the anterior end of the preserved palatine coincided with the area of the suture with the maxilla (Fig. 4A). It can thus be deduced that the palatine does not extend beyond the anterior margin of the suborbital fenestra and has a rounded shape similar to other basal crocodyloids such as *Prodiplocynodon*, “*Crocodylus” affinis* and *Brachyuranochampsa* [51]. A straight midline suture joins the two palatines; they do not reach the posterior end of the suborbital fenestra due to the intrusion of the pterygoid. The prefrontal pillar contacts the dorsal surface of the palatine, roughly in the area where the palatine begins its anterolateral expansion.

The maxillary ramus of the ectopterygoid is adjacent to the tooth row, almost contacting the final four or five maxillary alveoli (Fig. 4A) and possibly even forming the medial wall of the final two alveoli. The anterior part of the maxillary ramus ends in a point. The posterolateral process of the ectopterygoid contacts the lateroventral surface of the pterygoid, although the process does not reach as far as the posterior margin of the pterygoid. The ectopterygoid extends partially along the postorbital bar.

The pterygoids contact the palatines by means of a “zig-zag” suture on the posterolateral margin of the suborbital fenestra, but not in its most posterior angle. As in all eusuchians, the secondary choana is completely included within the pterygoids. The choana is located close to the posterior margin. The pterygoid is not depressed at the margins of the choana, and this does not form a neck. No septum divides the choanal opening. The pterygoid wings are dorsoventrally slender and are expanded posteriorly on the lateral margin where this articulates with the ectopterygoid. The posterior axis of the pterygoid is strongly curved, and a concave edge is formed in the posterior area of contact between the two pterygoids. The posteromedial process of the pterygoid is prominent and projects ventrally (Fig. 4B), displaying the plesiomorphic condition seen in other basal crocodyloids such as *Brachyuranochampsa eversolei*, *“Crocodylus” acer*, *“Crocodylus” affinis*, *Asiatosuchus germanicus* and *Prodiplocynodon langi* [51], [52]. In lateral view, sutures between the pterygoid, quadrate, and laterosphenoid are difficult to make out due to poor preservation, but a significant ventral process of the quadrate on the lateral braincase wall, together with the laterosphenoid, prevents the pterygoid from participating in the opening of the foramen ovale (Figs. 3B, 4A).

As in most crocodylians, with the exception of most alligatorids, the quadrate forms the anterior, ventral and posterior margin of the otic opening, and the squamosal forms the dorsal margin; in other words, the quadratosquamosal suture extends dorsally along the posterior margin of the opening. The dorsal surface of the quadrate lacks ornamentation. A gentle medial depression on the quadrate could correspond to the foramen aërum. The medial hemicondyle has not been preserved. Ventrally the quadrate is crossed by a crest associated with depressed areas, muscular insertion crest “B”, and though less marked, crest “A” also seems to be preserved (sensu [53]). The quadrate forms the posterolateral margin of the foramen ovale, which is surrounded by two longitudinal grooves, one aligned lateromedially and the other anteroposteriorly (Fig. 4A).

The neurocranium is the worst-preserved part of ELI-1. All that remains are the laterosphenoid, part of the basioccipital and part of the basisphenoid. The basisphenoid is very incomplete, but forms a thin sheet ventral to the basioccipital (Fig. 4). The right half of the basioccipital bone below the foramen magnum has been preserved (the occipital condyle is not preserved). This portion of the basioccipital is semicircular in posterior view, with ventral curvature, and it projects perpendicular to the occipital plane (Fig. 4B). The suture with the exoccipital is located just below the foramen magnum, slightly above where the quadrate begins. The foramen magnum is not well preserved. The laterosphenoid is Y-shaped, with three diverging rami, one projecting anteriorly and the other two posteriorly (Fig. 4A). These posterior rami participate in the anterolateral and anteromedial margin of the foramen ovale, where it makes contact with the quadrate. These rami of the laterosphenoid participate slightly in the posteromedial region of the posterior wall of the supratemporal fenestra, until the rami meet the ascending process of the quadrate, which occupies most of the posterior wall. The anterolateral ramus forms the base of the medial wall of the supratemporal fenestra, and wedges the parietal laterally. The capitate process of the laterosphenoid is oriented anteroposteriorly toward the midline.

### Phylogenetic relationships

**Figure 5:**
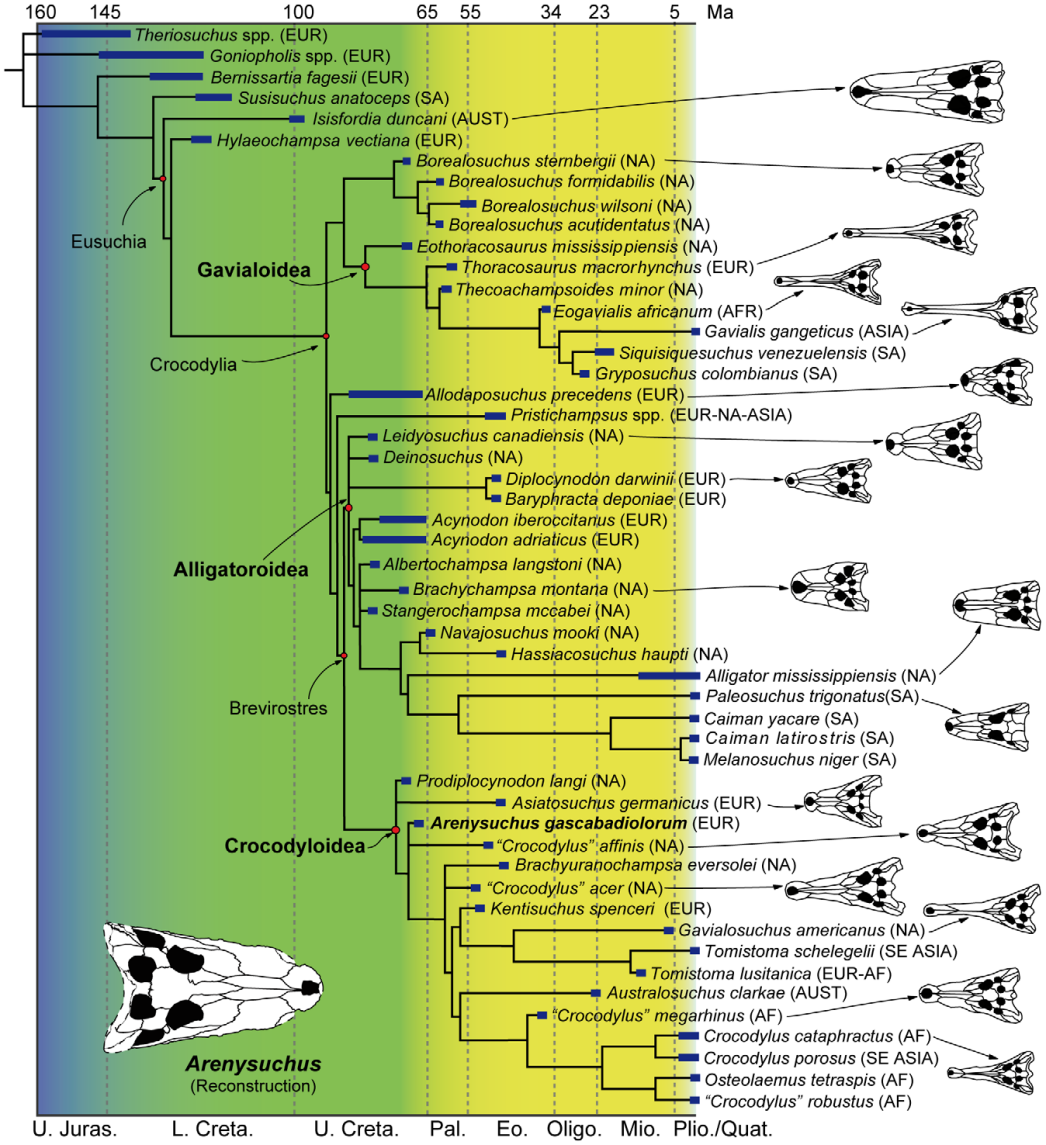
Phylogenetic relationships of *Arenysuchus gascabadiolorum*. Stratigraphically calibrated strict consensus of 368 equally parsimonious trees resulting from parsimony analysis of 176 characters in 51 taxa. Thick blue lines represent known minimal ranges (see [3], [5] and bibliographies therein). The abbreviations above the names denote the area in which the taxon occurs (AF, Africa; ASIA, Asia; AUST, Australia; EUR, Europe; NA, North America; SA, South America; SE ASIA, southeast Asia). For more information see Appendix S1 and Appendix S2 (data matrix, analysis protocol, apomorphy list, strict consensus tree and decay index).

The phylogenetic analysis places *Arenysuchus* in Crocodyloidea, as one of the most basal members of this clade (Fig. 5). In this position *Arenysuchus* appears in a polytomy with *“Crocodylus” affinis*, and as a sister group to *Brachyuranochampsa eversolei*, *“Crocodylus” acer* and the Crocodylidae family. *Prodiplocynodon langi* and *Asiatosuchus germanicus* are the taxa basal to the clade formed by *Arenysuchus*, *“Crocodylus” affinis*, *Brachyuranochampsa eversolei*, *“Crocodylus” acer* and Crocodylidae.

The base of Crocodyloidea is fairly poorly resolved in this analysis (three pairs of taxa forming polytomies in the strict consensus tree), and relationships among these taxa are weakly supported, as indicated by low decay indices (Appendix S1). This problem arises with the addition in the same analysis of the North American *“Crocodylus” affinis* and the European taxa *Asiatosuchus* and *Arenysuchus*, and this part of the cladogram appears well resolved when we do not include one of these taxa. When *Arenysuchus* is not included in the analysis this part of the cladogram appears as shown in Salisbury *et al.* [3], but *Prodiplocynodon* is basal to *Asiatosuchus* and they do not form a polytomy. When *Asiatosuchus* is not included, *Arenysuchus* appears as a descendant of *Prodiplocynodon* and basal to *“Crocodylus” affinis* and the other crocodyloids. And when *“Crocodylus” affinis* is not included, *Prodyplocinodon* and *Asiatosuchus* appear as an ancestral sister group of *Arenysuchus* and the other crocodyloids.

This phylogenetic position is supported by a series of characters. The palatine process of *Arenysuchus* does not extend beyond the rostral end of the suborbital fenestra, as is also the case in other basal crocodyloids, such as *Prodiplocynodon langi*, *Asiatosuchus germanicus*, “*Crocodylus” affinis* or “*Crocodylus” acer*. The basisphenoid is not broadly exposed ventral to the basioccipital in occipital aspect, as in most crocodyloids, with the exception of some such as *Prodiplocynodon*. Regarding the main differences among the taxa to which *Arenysuchus* is most closely related, we can pinpoint the several characters. As in most crocodylians, *Arenysuchus* presents a concavo-convex frontoparietal suture, but this suture enters the supratemporal fenestra, and the frontal thus prevents broad contact between the postorbital and the parietal, as happens in other basal crocodylians such as *Thoracosaurus*, *Leidyosuchus* and *Borealosuchus*. Unlike the other basal crocodyloids to which it appears to be closely related, *Arenysuchus* presents elevated dorsal rims of the orbits, a character also presented by more derived taxa.

There are some characters that diagnose *Arenysuchus* as a crocodyloid, and these distinguish it from most alligatoroids. In *Arenysuchus* the ectopterygoid abuts the maxillary tooth row; in alligatoroids the maxilla separates the ectopterygoid from the posterior maxillary tooth row. The foramen aërum is on the dorsomedial angle of the quadrate; in alligatoroids this foramen is located dorsally rather than dorsomedially. The fourth dentary tooth occludes in a notch between the premaxilla and the maxilla; in most alligatoroids this dentary tooth occludes in a closed pit. The lateral edges of the palatines are parallel caudally; in most alligatoroids the lateral edges of the palatines flare caudally, producing a shelf. The parietal and the squamosal are widely separated by the quadrate on the caudal wall of the supratemporal fenestra; in most alligatoroids the parietal and the squamosal approach each other or meet along the caudal wall of the supratemporal fenestra. *Arenysuchus* has secondary choanae that are not septate and are projected posteroventrally; in most alligatoroids the choanae are septate and projected anteroventrally. The fifth maxillary alveolus is the largest; in most alligatoroids the fourth maxillary alveolus is the largest, or the alveoli are homodont.

The relationships between the taxa considered in the analysis are consistent with previous morphological analyses, with some exceptions such as the weakly supported sister relationship between *Borealosuchus* and Gavialoidea [3], *Borealosuchus* being a descent group of Gavialoidea in other analyses [5], [12], [44], [54], [55]. Another phylogenetic controversy arises from adding the European taxa *Acynodon adriaticus* Delfino, Martin and Buffetaut 2008, *Acynodon iberoccitanus* Buscalioni, Ortega and Vasse 1997 and *Allodaposuchus precedens* to the analysis. The matrix scores of *Acynodon* [56] in this analysis place this taxon as a basal globidontan and one of the oldest alligatoroids, which is consistent with previous phylogenetic analyses [10], [14], [56]. The addition of the new specimen of *Allodaposuchus* cf. *A. precedens* [16] recovered from France changes the phylogenetic position of this species with respect to previous studies. The first phylogenetic analyses placed *Allodaposuchus* at the base of Eusuchia [12], [57], probably as a result of the incomplete material. Moreover, the specimens included in the matrix stemmed from various European localities and might have belonged to different taxa [3], [16]. The paper by Martin [16], which is based on very complete material, places it at the base of Alligatoroidea, and suggests that *Allodaposuchus precedens* might also be a basal globidontan. These discrepancies are mainly due to differing interpretations of certain characters. As this paper has used the *Allodaposuchus* characters provided by Martin [16], the position of this taxon is similar to that in his analysis, though in our case the genus is situated at a somewhat more basal node (Fig. 5), as a sister taxon to *Pristichampsus* and Brevirostres. Nevertheless, a more recent study indicates that neither of these taxa are crocodylians, and *Acynodon* (which may not be monophyletic) and *Allodaposuchus* could be part of an endemic European radiation (Hylaeochampsidae) [55].

## Discussion

### Palaeobiogeographic and phylogenetic implications

During the Maastrichtian, Europe was divided into a set of islands that formed an archipelago of great palaeobiogeographical and evolutionary interest. Between North America and the European archipelago, palaeogeographical bridges sporadically connected the two continents and permitted faunal exchange between them [58]–[61]. Moreover, the layout of Europe as an archipelago fostered endemism and vicariant evolution in terrestrial vertebrates such as crocodylomorphs and dinosaurs [4], [62]–[64].

There are two main distinct patterns in the spatial distribution of crocodylomorphs in the Late Cretaceous: Europe/North America and Africa/Madagascar/South America/Asia. In Europe and North America a high proportion of genera of Crocodylia have been described, exceeding 50 percent of the total number of genera. In Asia, South America, Africa and Madagascar, by contrast, Crocodylia represents less than 15 percent of the total number of genera, with non-crocodylians dominating [1]. An interpretation for this might be that Crocodylia underwent radiation in Europe and North America towards the end of the Late Cretaceous (Campanian-Maastrichtian) [1], [3], [4], [65].

The radiation of Crocodylia in Europe and North America during the Late Cretaceous may have been a consequence of the same causes that gave rise to the faunal turnover among other vertebrates in the Campanian-Maastrichtian interval. This turnover has been attributed to a climatic change produced by a marine regression in Europe during the Maastrichtian [23], [59], [66]–[70]. The European mesoeucrocodylian fauna was exclusively non-crocodylian in the Turonian-Santonian, yet in the Campanian and above all in the Maastrichtian the non-crocodylian genera seem to disappear, and crocodylians become an increasingly important part of the fauna, equalling them in number of genera in Europe and eventually outnumbering them in North America [1]. In Europe, this faunal turnover can also be noted in other groups of vertebrates, such as dinosaurs. The dinosaurs of the Campanian and early Maastrichtian of western Europe are relatively abundant and well known. Titanosaurian sauropods, ornithopods such as *Rhabdodon*, ankylosaurians, and theropods such as dromaeosaurids are represented [58], [71], [72]. This situation changes in the late Maastrichtian, where the dinosaur remains are scarce and fragmentary, consisting almost exclusively of hadrosaurids [73]–[77]. The faunal turnover that affected the dinosaurs might also have affected the crocodylomorphs.

In North America and Europe, one finds the basal members of Crocodylia, such as *Borealosuchus*, the crocodyloids *Prodiplocynodon* and *Arenysuchus*, and the alligatoroids *Brachychampsa*, *Stangerochampsa*, *Leidyosuchus*, *Deinosuchus*, *Acynodon*, *Musturzabalsuchus*, *Allodaposuchus* and *Albertochampsa*. However, there is no consensus regarding the phylogeny of certain European basal members of Alligatoroidea, since some authors place *Allodaposuchus* as basal eusuchians outside Alligatoroidea and Crocodylia [12], [55], [57], whereas other papers have placed it at the base of Alligatoroidea [16]. The same applies to *Acynodon*; some authors place it as a basal globidontan alligatoroid [10], [14], [56], while more recent studies place it outside Crocodylia (as an endemic hylaeochampsid) [55]. The record of gavialids seems more widely dispersed and includes the southern hemisphere, but this is due to its marine lifestyle, which enabled it to undertake large migrations between landmasses. In any case, alligatoroids display the greatest degree of diversification in the Late Cretaceous, forming more than 50 percent of described genera [1]. By contrast, the fossil record of Crocodyloidea is scarce in the Late Cretaceous, comprising only *Prodiplocynodon langi* of the Lance Formation (Maastrichtian) in Wyoming (USA) [51]. Members of Crocodyloidea are cited in Laurasia from the Palaeogene onwards, with *Asiatosuchus* [52] of the Palaeocene of Europe [78], [79] and Asia [80] being the oldest known representative up to now. Cladistic analyses place *Arenysuchus* within Crocodyloidea, making it the oldest known crocodyloid from Europe.

The continental associations of crocodylomorphs in North America and Europe were well diversified during the Late Cretaceous due to vicariance [10]; in other words, they did not share genera between continents. This tendency continued throughout the Palaeocene and Eocene, with few genus-level similarities in the continental crocodylian faunas of North America and Europe during the Cenozoic [81]. The only common genera between continents were coastal and marine ones: for example, dyrosaurids appeared in Europe and Africa; *Hyposaurus* in North America and South America; and the gavialid *Thoracosaurus* appeared in North America, Europe, Asia and Africa [1].

We performed a palaeobiogeographic cluster analysis (Appendix S4). The analysis shows a clear relation between the crocodylian fauna of North America, the Iberian Peninsula and the rest of Europe, which has little in common with the non-crocodylian fauna predominant in the other continents. The joint presence of Crocodylia in Europe and North America during the Maastrichtian can be explained by the existence of land bridges. Such dispersal has been cited in other groups of vertebrates, such as marsupials, theropod and hadrosaurid dinosaurs, and boa serpents (Boidae), which appear both in European and North American sites of the end of the Cretaceous [59], [82], [83]. The dispersal route of these faunas would have been the “Thulean Land Bridge” [84], [85]. This land bridge would have connected the islands of the northeast of Canada, Greenland and the British Isles, from which there would have been access to the rest of Europe. Bearing in mind the aquatic or semi-aquatic nature of most crocodylians, they could have used routes other than the exclusively terrestrial ones, albeit associated with the shallow seas that were abundant in the North Atlantic at the end of the Cretaceous.

## Materials and Methods

### Fossil preparation

ELI-1 was prepared using formic acid. The fossil, within the matrix, was immersed for two to three days in a vessel containing a solution of formic acid diluted to between 5% and 8% in water. The acid was renewed as it became saturated. The parts of the fossil without matrix were strengthened and protected with Paraloid (acrylic resin) prior to being immersed in the acid. After two or three days in the acid, the salts that had formed were removed by means of gentle water baths in order to avoid breaks in the newly exposed parts. The specimen was left to dry for at least a day in order to prevent white patinas forming during the consolidation phase as a result of moisture in the fossil. The process was repeated three times until the fossil and the matrix were completely separated.

### Phylogenetic methodology

The list of characters and taxa used in this paper is the same as in Salisbury *et al*. [3] The following scores in this matrix have been updated after a personal communication by S. Salisbury: *Isisfordia*; character 69, changed from 1 to 0 (quadratojugal spine present). *Australosuchus clarkei*; character 118, changed from 0 to 1 (wedged-shaped rostral process of the palatine). In addition, we have added the taxa *Arenysuchus*, *Acynodon adriaticus* and *Acynodon iberoccitanus* [56], *Albertochampsa langastoni* Erickson 1972 and *Deinosuchus* (scores provided by S. Salisbury) and replaced *Allodaposuchus precedens* [12] by a new and more complete specimen of *Allodaposuchus* cf. *A. precedens* [16] (see Appendix S1 and Appendix S2). The matrix of characters was analysed using the application PAUP 4.0b10 [86]. A total of 176 characters were analysed for 51 taxa. The taxa *Goniopholis* and *Theriosuchus* were defined as an outgroup to root the trees. The analysis was completed with unordered multi-state characters, except for characters 18, 36, 170 and 171, which were ordered so that character transformations were compatible with the most recent biomechanical analyses [3], [87]. Decay index analysis (Bremer support) was performed for each node with TNT 1.1 [88] (Appendix S1).

Ten heuristic searches were completed, using Random Stepwise Addition in each case, and 368 optimal trees of maximum parsimony were obtained (tree length = 534 evolutionary steps; consistency index (CI) = 0.4251; homoplasy index (HI) = 0.5749; CI excluding non-informative characters = 0.4085; HI excluding non-informative characters = 0.5915; retention index (RI) = 0.7446; rescaled consistency index (RC) = 0.3165). On the basis of the 368 maximally parsimonious trees a chronostratigraphically calibrated consensus tree was constructed using the Strict method (Fig. 5). The chronostratigraphic calibration is based on the work of Salisbury *et al.* [3].

### Palaeobiogeographic methodology

We performed a multivariate cluster analysis for the Late Cretaceous crocodylomorphs (paired group and Dice distance, cophenetic correlation = 0.7765) with PAST 1.94b software [89], using 19 taxa and 10 palaeobiogeographic provinces (Appendix S4).

## Supporting Information

Appendix S1 Supplementary phylogenetic information, including data matrix, taxa list (see strict consensus tree), analysis protocol, strict consensus tree, complete list of apomorphies of the nodes present in the strict consensus tree, and decay index for each node.
(DOC)

Appendix S2 NEXUS file of the data matrix used in the phylogenetic study.
(NEX)

Appendix S3 SEM photographs of teeth found in Blasi 2 site.
(DOC)

Appendix S4 Palaeobiogeographic cluster analysis.
(DOC)

Appendix S5 Skull measurements.
(DOC)
