# Correction: A New Crocodylian from the Late Maastrichtian of Spain: Implications for the Initial Radiation of Crocodyloids

**DOI:** 10.1371/annotation/c62bd5eb-d95d-44f5-946c-28ade9c4cd44

**Published:** 2011-06-30

**Authors:** Eduardo Puértolas, José I. Canudo, Penélope Cruzado-Caballero

The Funding section is incomplete. The complete funding section should read: "This paper forms part of the projects CGL2007-62469 and CGL2010-16447, subsidized by the Ministry of Science and Innovation, the European Regional Development Fund, the Government of Aragon ("Grupos Consolidados" and "Dirección General de Patrimonio Cultural") and the "Instituto de Estudios Altoaragoneses". The funders had no role in study design, data collection and analysis, decision to publish, or preparation of the manuscript."

