# Supplementary material for: A New Crocodylian from the Late Maastrichtian of Spain: Implications for the Initial Radiation of Crocodyloids
Source: PLoS One. 2011 Jun 8;6(6):e20011. doi: 10.1371/journal.pone.0020011 (PMC3110596; doi:10.1371/journal.pone.0020011)
Supplement: Appendix S1 — Supplementary phylogenetic information, including data matrix, taxa list (see strict consensus tree), analysis protocol, strict consensus tree, complete list of apomorphies of the nodes present in the strict consensus tree, and decay index for each node. (DOC) [file pone.0020011.s001.doc]

**Supporting Information – Appendix S1**

**A New Crocodile from the Upper Maastrichtian of Spain: Implications for the Initial Radiation of Crocodylids.**

**Eduardo Puértolas^1^, José I. Canudo^1^, Penélope Cruzado-Caballero^1^**

^1^Grupo Aragosaurus-IUCA (www.aragosaurus.com), Paleontología, Facultad de Ciencias, Universidad de Zaragoza, C/ Pedro Cerbuna 12, 50009 Zaragoza, España.

A total of 176 characters were analysed for 51 taxa. The taxa Goniopholis and Theriosuchus were defined as an outgroup to root the trees. Ten heuristic searches were completed, using Random Stepwise Addition in each case, and 368 optimal trees of maximum parsimony were obtained (tree length = 534 evolutionary steps; consistency index (CI) = 0.4251; homoplasy index (HI) = 0.5749; CI excluding non-informative characters = 0.4085; HI excluding non-informative characters = 0.5915; retention index (RI) = 0.7446; rescaled consistency index (RC) = 0.3165).

**Data matrix**

The list of characters and taxa used in this paper is the same as in Salisbury *et al*., [3] (The following scores in this matrix have been updated after a personal communication by S. Salisbury: Isisfordia; character 69, changed from 1 to 0 (quadratojugal spine present). Australosuchus clarkei; character 118, changed from 0 to 1 (wedged-shaped rostral process of the palatine). In addition we have added the taxa *Arenysuchus*, *Acynodon adriaticus* and *Acynodon iberoccitanus* [Delfino et al., 2008a], *Albertochampsa langastoni* Erickson 1972 and *Deinosuchus* (scores provided by S. Salisbury) and the replacement of *Allodaposuchus precedens* [12] by a new and more complete specimen of *Allodaposuchus cf. A. precedens* [15]. The full matrix is also available in nexus file (Appendix S2).

*Arenysuchus gascabadiolorum*: ?????????? ?????????? ?????????? ?????????? ?????????? ?????????? ?????????1 1001010110 0??0110110 01001??000 0?100?0001 ??1???0000 0?10??0??1 00010?1?01 ?00001??1? ?01??????? 0?0?0??1?? ?1?111

*Allodaposuchus cf. precedens* ?????????? ?????????? ?????????? ?????????? ?????????? ?????????? ?????????0 1101000?10 02110?1020 01001?00?0 00000??0?1 01???000?0 ??11000??? 10010???00 000101??10 1?1??????? 1???0????? ?1?111

*Acynodon iberoccitanus* ?????????? ?????????? ?????????? ?????????? 1?1??2???? ??1100??0? ?0?NN??010 1?0?021010 0?00010130 11201?01?0 01001?00?0 000101000? ??10000?0? 21010???01 1000111010 ??1??????? 000?101??? ?1??11

*Acynodon adriaticus* ?????????? ???????2?? ?100?????? ????01???0 ??1?????10 0?????00?? ?0?NN????0 100?0?1?0? 0??0110130 ???01?0??0 0?0??000?0 ??????00?? ??1??????? ????0???01 1000?1???? ?01??????? 0???1????? ?1??11

*Albertochampsa langstoni* ?????????? ?????????? ?????????? ?????????? ?????????? ?????????? ?????????1 110?0?101? 1000010121 1120??00?0 01000?00?? 001??0001? 1?1??0?0?? 2??10???01 1?00110??0 01???????? 0???001??? ?1?111

*Deinosuchus* ?????????? ???????2?? ?????????? ????12???1 ??0??01111 ?011?0???? ?1?00??001 ???022?0?0 0011?10??? ?1???????0 012000??0? ?1?10?0??0 1?1???0??0 0101011001 1?00?1???0 ?????????? 000??????? 3???1?

**Apomorphy list**

The tree used to derive these apomorphies is identical to the one shown in Fig. 5.

* = ACCTRANS optimisation only.

** = DELTRANS optimisation only.

*Bernissartia fagesii* + *Susisuchus anatoceps* + Eusuchia: 7(2), **36(1), **37(1), **76(1), **85(1), **175(1).

*Bernissartia fagesii*: 3(1), 4(1), 12(1), *36(1), *37(1), 53(0), *76(1), *85(1), 91(1),105(1), 113(1), 152(0), 171(3), *175(1).

*Susisuchus anatoceps* + Eusuchia: 36(1), *37(1), **50(1), *76(1), **79(1), **92(1), **141(1), *175(1).

*Susisuchus anatoceps*: *36(2), 38(3), *50(1), 69(1), 77(1), *79(1), 82(2), 89(3), *92(1), 93(2), 131(2), *141(1), 146(0), 169(1), 171(0).

Eusuchia: **18(1), *36(2), **62(1), **71(1), *92(1), *141(1), **149(1), **170(2), **171(2).

*Isisfordia duncani*: 9(0), 11(1), 17(1), *18(1), 28(1), 38(1), *62(1), *71(1), 94(1), *149(1), 167(3), *170(2), *171(2).

*Hylaeochampsa vectiana* + Crocodylia: *71(1), *85(1), **113(1), **119(1), **130(1), **152(0), **172(1), **174(1).

*Hylaeochampsa vectiana*: 103(2), *113(1), 117(1), *119(1), 123(0), *130(1), 141(0), 146(0), *152(0), *172(1), *174(1).

Crocodylia: 18(1), *50(1), *62(1), **86(1), **93(0), *113(1), *149(1), *152(0), **168(1), *170(2), 171(2), *172(1), **173(1), *174(1).

Gavaloidea + *Borealosuchus*: 7(0), 10(0), 14(0), *18(2), 19(1), 35(1), 37(0), *39(2), *58(1), 60(0), *61(0), 78(2), *86(1), *93(0), *118(1), 140(1), *168(1), *171(3), *173(1).

*Borealosuchus*: 11(1), 12(1), 29(1), 51(0), 70(1), **79(0), *119(1), *130(1), 135(1), 137(1), 169(1).

*Borealosuchus formidabilis* + *Borealosuchus wilsoni* + *Borealosuchus acutidentatus*: **39(2), *61(1), 86(0), 117(2), **118(1).

*Borealosuchus formidabilis*: **58(1), 167(3), 173(0).

*Borealosuchus wilsoni* + *Borealosuchus acutidentatus*: *43(1), *45(2), 81(1), 166(1).

*Borealosuchus wilsoni*: **43(1), **45(2).

*Borealosuchus sternbergii*: 17(1), *58(0), **61(0), 78(1), 88(1), 94(1), *118(0), 120(1), 134(1).

Gavialoidea: *2(2), *3(1), *4(1), *9(0), *27(1), *30(1), 33(1), *39(3), 43(3), *52(1), 68(2), *79(1), 84(1), 89(3), **118(1), **119(0), 123(0), 145(1), 146(0), *153(0), 166(2), 167(3).

*Thoracosaurus macrorhynchus* + *Thecoachampsoides minor* + *Eogavialis africanum* + *Gavialis gangeticus* + *Siquisiquesuchus venezuelensis* + *Gryposuchus colombianus*: **3(1), **4(1), **52(1), 103(1), **153(0), *174(0).

*Thecoachampsoides minor* + *Eogavialis africanum* + *Gavialis gangeticus* + *Siquisiquesuchus venezuelensis* + *Gryposuchus colombianus*: **27(1), 81(1), 113(0), 151(1).

*Thecoachampsoides minor*: 112(3), *130(1).

*Eogavialis africanum* + *Gavialis gangeticus* + *Siquisiquesuchus venezuelensis* + *Gryposuchus colombianus*: *35(0), 103(2), **130(0), 139(1).

*Gavialis gangeticus* + *Siquisiquesuchus venezuelensis* + *Gryposuchus colombianus*: **61(0), 86(0), 93(1), 98(2), **174(0).

*Siquisiquesuchus venezuelensis* + *Gryposuchus colombianus*: *74(1), 140(2).

*Siquisiquesuchus venezuelensis*: 23(1).

*Gryposuchus colombianus*: 54(1), **74(1).

*Gavialis gangeticus*: **2(2), **9(0), **30(1), **35(0), **39(3), **58(1), 84(0), 95(3).

*Eogavialis africanum*: 20(1), 47(1), 60(1), 82(1).

*Thoracosaurus macrorhynchus*: 86(0), 116(1), **130(0).

*Allodaposuchus cf. precedens + Pristichampsus* spp. + Brevirostres: *79(1), *93(0), **95(1), **134(1).

*Allodaposuchus cf. precedens*: 72(1), 74(1), 76(0), 82(2), 83(1), 84(1), 85(0), 87(1), 89(2), *95(1), 110(1), 112(1), 124(1), 131(1), *134(1), 141(0), 144(1), 151(1), 161(1).

*Pristichampsus* spp. + Brevirostres:7(0), *18(2), **27(1), **29(1), **33(1), **70(1), *86(1), *95(1), *119(1), *130(1), *134(1),**140(1), *171(3), *173(1).

*Pristichampsus* spp.: 12(1), *27(1), 28(1), *29(1), *33(1), 45(1), 52(1), *70(1), 79(0), 81(2), 103(1), 112(2), 128(1), 132(1), *140(1), 162(1), 165(1).

Brevirostres: **8(2), *12(1), **20(1), **26(1), *27(1),*29(1), *33(1), **34(1), **54(1), *70(1), **88(1), **102(1), *112(1), *132(1), **136(1), **137(1), *140(1), **160(1), **164(1), **167(0), *168(1), **169(1).

Alligatoroidea: 1(1), *8(2), **12(1), *20(1), *26(1), *34(1), 38(1), 48(1), 49(1), *54(1), *77(1), *88(1), 91(1), *102(1), **112(1), 114(1), 121(1), **132(1), *136(1), *137(1), *155(1), *156(1), *159(1), *160(1), *164(1), *167(0), *169(1).

*Leidyosuchus canadensis*: 40(1), 54(0), *77(0), 79(0), 94(1), 105(1), 132(0), 136(0).

Diplocynodontidae: 6(0), 10(0), 28(4), 39(2), *45(1), 69(1), **77(1), 86(0), 90(1), *95(2), *131(1).

*Baryphracta deponiae*: 87(1).

*Diplocynodon darwinii:* 43(1), **45(1), 79(0), **95(2), **131(1).

*Deinosuchus:* 35(1), 40(1), 47(1), 75(2), 76(2), 83(1), 84(1), 103(2), 130(0).

Globidonta: *5(1), *41(1), *42(1), *52(1), *66(1), 69(1), *74(1), 76(2), **77(1), *85(0), *89(1), 93(2), 131(2), 145(1), 167(1).

*Acynodon*: *22(1), *36(1), 43(1), *46(2), *50(0), *51(0), 62(0), 70(0), 89(3), *98(1), *105(1), *112(0), *113(0), *116(1), *119(0), *147(1), 165(1).

*Acynodon adriaticus*: **22(1), **36(1), **50(0), **51(0), 79(0), *85(1).

*Acynodon iberoccitanus:* **41(1), **46(2), **85(0), **98(1), **105(1), **112(0), **113(0), **116(1), **119(0), **147(1).

*Stangerochampsa mccabei*: 19(1), 35(1), *41(0), **42(1), 65(1), **66(1), **124(2).

*Brachychampsa Montana*: 11(1), 37(3), **41(1), *42(0), 43(1), 82(2), 89(1), 108(1), **124(2), 131(1).

*Albertochampsa langstoni*: 112(0).

*Navajosuchus mooki* + *Hassiacosuchus haupti* + *Alligatoridae*: 17(1), 21(1), *25(1), *28(1), **42(1), 81(2), *82(1), 93(1), *124(0), 163(1).

*Navajosuchus mooki* + *Hassiacosuchus haupti*: *1(0), *41(0), 68(1), 79(0), *124(1).

*Navajosuchus mooki*: 65(1), *82(0), **124(1).

*Hassiacosuchus haupti*: **1(0), **82(1).

Alligatoridae: *22(1), **25(1), **28(1), *39(2), **41(1), 43(2), **59(1), 86(0), 103(1), 145(0), **155(1), **156(1), **159(1), 166(1), *167(0).

*Alligator mississippiensis*: **22(1), 23(1), 37(2), 39(3), 45(1), 64(1), *66(0), **82(1), 95(0), 99(1), 106(1), 115(1), 117(1), 142(1), 152(2), **167(0).

Camininae: 16(1), 24(1), *38(2), **39(2), *46(1), 47(1), 51(0), 55(1), 58(1), **66(1), 67(1), 80(1), *82(2), 87(1), *104(1), 107(1), 116(1), 151(2), *157(1), 167(2).

*Caiman yacare* + *Caiman latirostris* + *Melanosuchus niger*: 11(1), 12(0), **22(1), **38(2), 44(2), **46(1), *54(0), 82(3), **104(1), 105(1), 153(2), **157(1).

*Caiman yacare*: 61(0), 78(1), 86(1), 100(1).

*Caiman latirostris* + *Melanosuchus niger*: 143(1), 156(2).

*Caiman latirostris*: 14(0).

*Melanosuchus niger*: 37(2), 125(1).

*Paleosuchus trigonatus*: 7(1), 10(0), 15(1), *22(0), 28(3), 30(2), 31(1), 38(3), 42(2), 46(2), 56(1), **82(2), 93(0), 94(1), 96(1), 97(1), 108(1), 157(2).

Crocodyloidea: 8(2), **17(1), *20(1), *26(1), *34(1), **51(0), 81(1), *88(1), *102(1), 110(1), 111(1), 112(3), **122(1), 128(1), *137(1), *167(0), *169(1).

*Prodiplocynodon langi*: *122(1).

*Asiatosuchus germanicus*: 3(1), *12(0), *17(1), *51(0), *52(1), 79(0), 86(0), 89(1), *132(0).

***Arenysuchus gascabadiolorum*** + *‘Crocodylus’ affinis* + *Brachyuranochampsa eversolei* + *‘Crocodylus’ acer* + Crocodylidae: *12(0), 13(1), *17(1), 28(1), *38(1), 41(1), 43(1), *51(0), *52(1), *54(1), **78(1), 81(2), 89(1), 119(0), *122(1), *132(0), *136(1), 154(1), *156(1), *160(1), 162(1), *164(1), 167(2).

***Arenysuchus gascabadiolorum*:** 74(1), *78(1), 81(0), 103(1).

*‘Crocodylus’ affinis*: 11(1), *78(1).

*Brachyuranochampsa eversolei* + *‘Crocodylus’ acer* + Crocodylidae: *3(1), *14(0), *25(1), *30(1), *39(3), *44(1), *45(1), 78(2), 111(0), *120(1), 127(1), *129(1), *138(1), 145(1), *158(1).

*‘Crocodylus’ acer*: 80(1).

*Brachyuranochampsa eversolei*: 86(0).

Crocodylidae: **3(1), **14(0), **25(1), **44(1), 83(1), 98(1), 103(1), 110(0), *118(1), **120(1), **129(1), **138(1), **156(1), **158(1).

*Australosuchus clarkae* + *‘Crocodylus’ megarhinus* + Crocodylinae: *1(1), *7(1), *22(1), *30(2), **45(1), *57(1), 75(2), 76(0), 80(1).

*Australosuchus clarkae*: 112(1), **118(1), 132(1).

*‘Crocodylus’ megarhinus* + Crocodylinae: *118(0), 145(0).

*‘Crocodylus’ megarhinus*: 9(0), 89(0), 93(2).

Crocodylinae: **22(1), **30(2), **38(1), *47(1), **57(1), 74(1), 75(1), *106(1), *144(1).

*Crocodylus*: **1(1), 28(2), **39(3), **47(1), **106(1), 109(1), 148(1).

*Crocodylus cataphractus*: 3(0), *7(0), 14(1), 61(0), 95(2), 118(1), *144(0), 167(3).

*Crocodylus porosus*: 2(1), **7(1), 25(0), 38(2), 40(1), **144(1), 147(1).

*Osteolaemus tetraspis* + *‘Crocodylus’ robustus*: 32(1), *39(1), 73(1), 87(1), 94(1), *96(1), 110(1), **144(1), 150(1), 163(1).

*Osteolaemus tetraspis*: **7(1), **47(1), 80(0), 86(0), 95(0), **96(1), 97(1), 105(1), *106(0), 119(1).

*‘Crocodylus’ robustus*: *47(0), 49(1), 82(1), **106(1).

*Kentisuchus spenceri* + *Gavialosuchus americanus* + *Tomistoma*: *2(2), *38(3), 43(4), *61(0), *68(2), 88(0), 93(1), **118(1), 167(3).

*‘Crocodylus’ spenceri*: **45(1).

*Gavialosuchus americanus* + *Tomistoma*: **2(2), *45(0), *60(0), **68(2), *84(1), 95(2).

*Gavialosuchus americanus*: 10(0), 17(0), 23(1), 45(2), 82(1), **84(1).

*Tomistoma*: *44(0), *54(0), 86(0), 126(1).

*Tomistoma schlegelii*: **30(1), **38(3), **39(3), **44(0), 47(1), **54(0), **60(0), **61(0), *84(0), 110(1), 118(0).

*Tomistoma lusitanica*: 80(1), **84(1), 93(0).

**Strict consensus tree and decay index (Bremer support):**

The color numbers correspond to the decay index for each node.

**
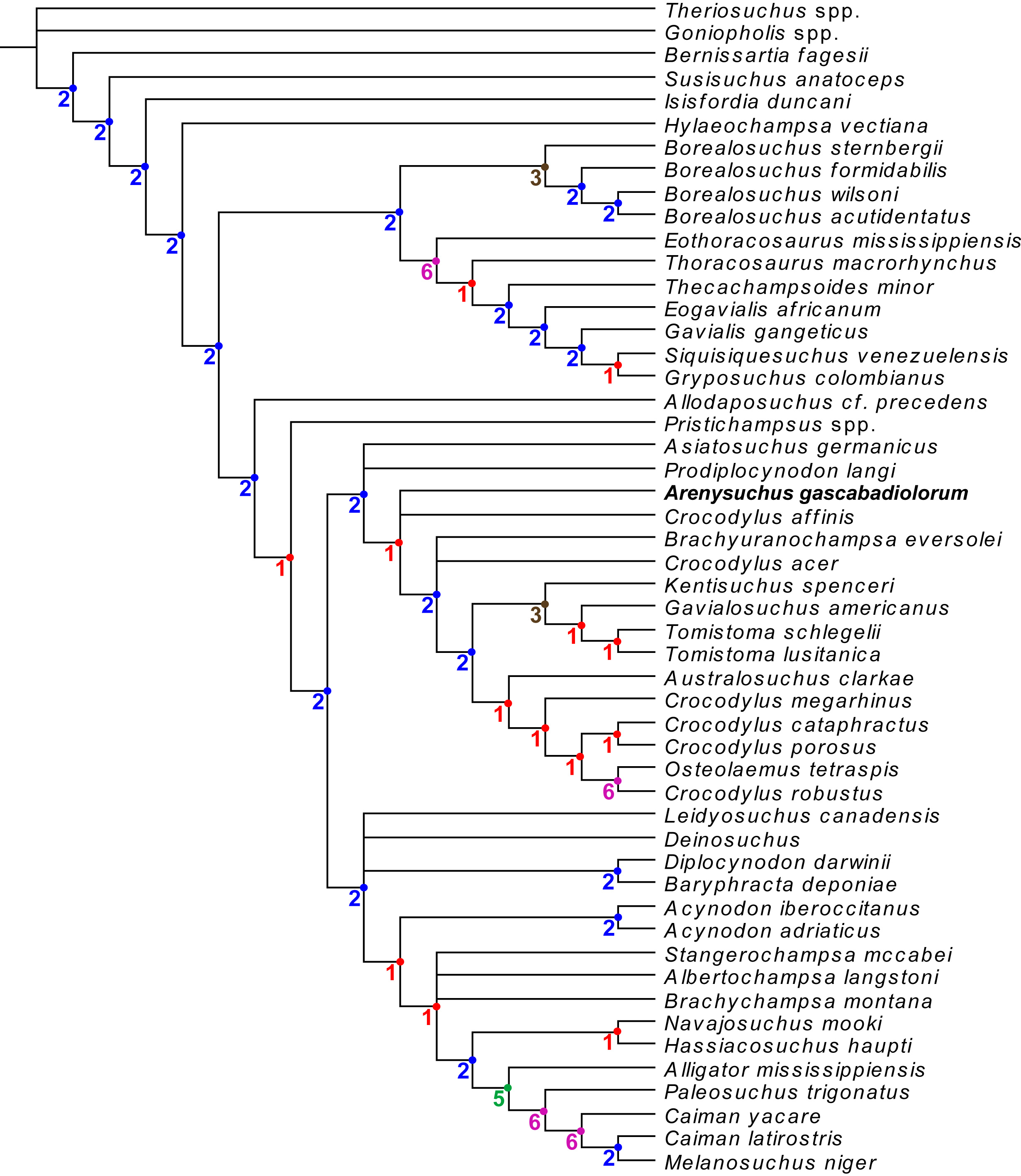
**
