# Supplementary material for: A New Crocodylian from the Late Maastrichtian of Spain: Implications for the Initial Radiation of Crocodyloids
Source: PLoS One. 2011 Jun 8;6(6):e20011. doi: 10.1371/journal.pone.0020011 (PMC3110596; doi:10.1371/journal.pone.0020011)
Supplement: Appendix S3 — SEM photographs of teeth found in Blasi 2 site. (DOC) [file pone.0020011.s003.doc]

**Supporting Information – Appendix S3**

**A New Crocodile from the Upper Maastrichtian of Spain: Implications for the Initial Radiation of Crocodylids.**

**Eduardo Puértolas^1^, José I. Canudo^1^, Penélope Cruzado-Caballero^1^**

^1^Grupo Aragosaurus-IUCA (www.aragosaurus.com), Paleontología, Facultad de Ciencias, Universidad de Zaragoza, C/ Pedro Cerbuna 12, 50009 Zaragoza, España.

**SEM photographs of teeth found in Blasi 2 site:**


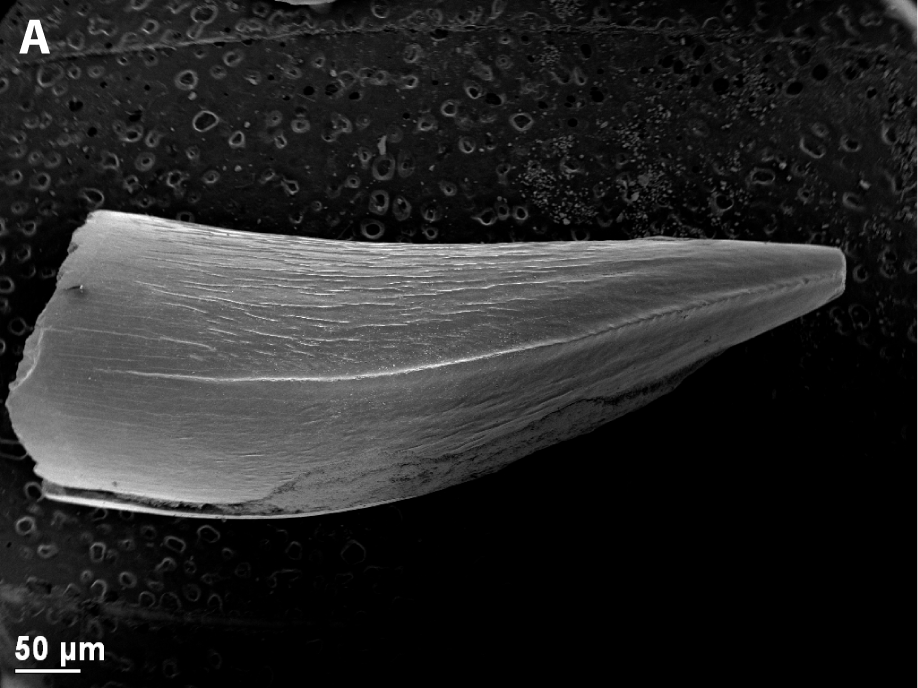


**Appendix S3.** A, Conical tooth MPZ2010/948 (in distal view) found in Blasi-2 site with similar morphology to the premaxillary tooth of *Arenysuchus*. This tooth has a conical shape, slightly curved lingually, with mesial and distal carinae well developed without denticles, and smooth ornamentation with ridges more developed in the lingual region.

**
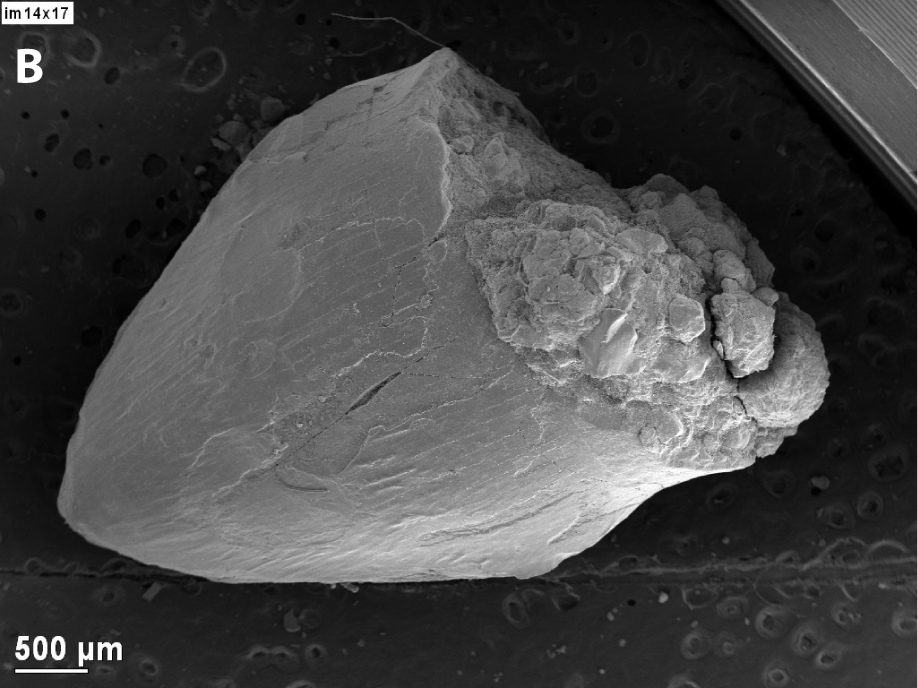
**

**Appendix S3.** B, Lanceolate tooth MPZ2010/949 (in lingual view) found in Blasi-2 site similar to the maxillary posterior teeth of *Arenysuchus*. This tooth has a lanceolate shape, is low, small in size, slightly flattened labiolingually, wider mesiodistally, with mesial and distal carinae without denticles, and with smooth ridges in the enamel.
