# Supplementary material for: A New Crocodylian from the Late Maastrichtian of Spain: Implications for the Initial Radiation of Crocodyloids
Source: PLoS One. 2011 Jun 8;6(6):e20011. doi: 10.1371/journal.pone.0020011 (PMC3110596; doi:10.1371/journal.pone.0020011)

**Supporting Information – Appendix S4**

**A New Crocodile from the Upper Maastrichtian of Spain: Implications for the Initial Radiation of Crocodylids.**

**Eduardo Puértolas^1^, José I. Canudo^1^, Penélope Cruzado-Caballero^1^**

^1^Grupo Aragosaurus-IUCA (www.aragosaurus.com), Paleontología, Facultad de Ciencias, Universidad de Zaragoza, C/ Pedro Cerbuna 12, 50009 Zaragoza, España.

**Palaeobiogeographic cluster analysis:**

Multivariate cluster analysis for the Upper Cretaceous crocodylomorphs (Paired group and Dice distance, cophenetic correlation=0.7765) with the software PAST 1.94b [80] which have been used 19 taxa and 10 palaeobiogeographic provinces. 0 and 1 indicate absence or presence of the taxa for each province.


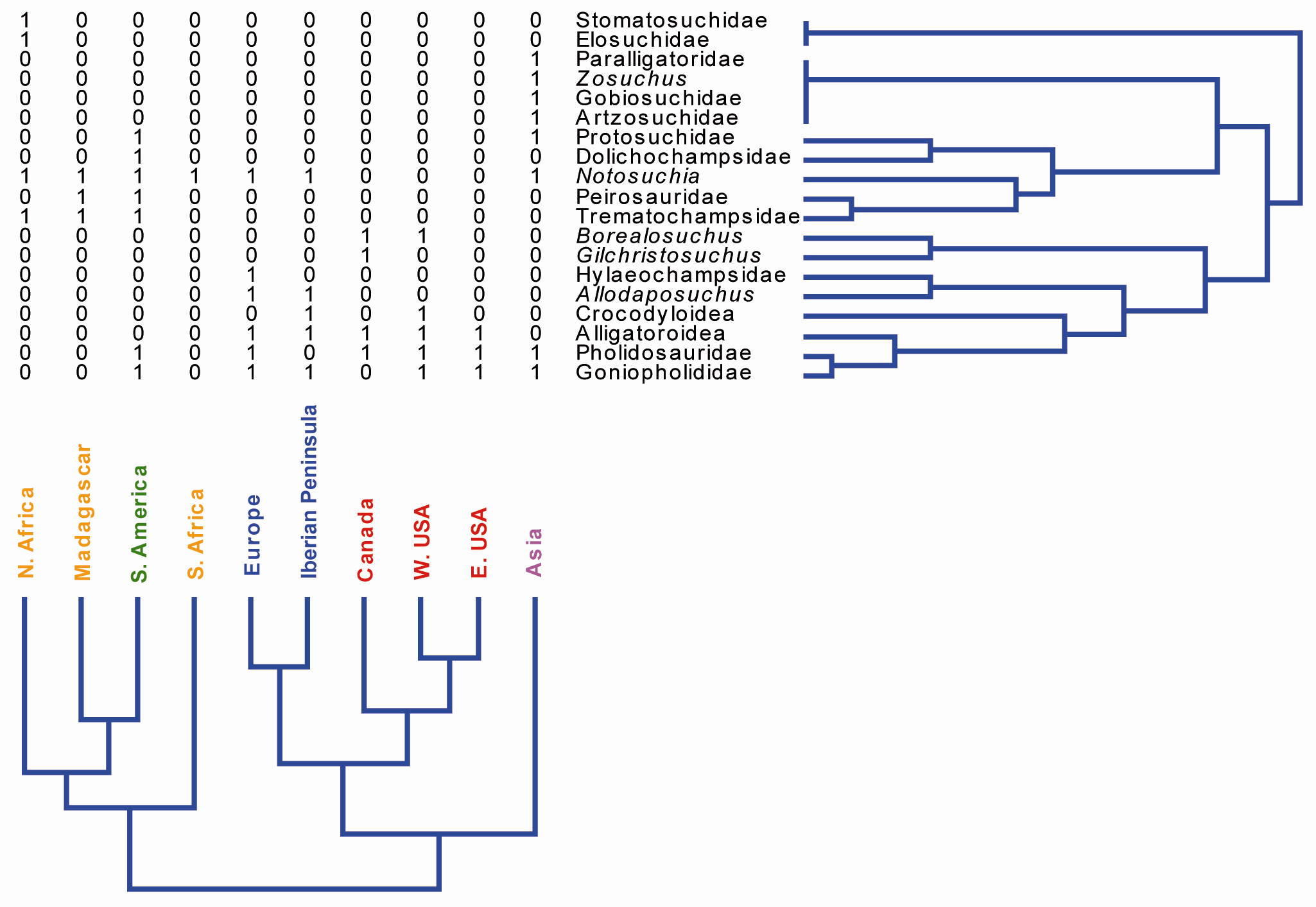

Supplement: Appendix S4 — Palaeobiogeographic cluster analysis. (DOC) [file pone.0020011.s004.doc]
