# Supplementary material for: A New Crocodylian from the Late Maastrichtian of Spain: Implications for the Initial Radiation of Crocodyloids
Source: PLoS One. 2011 Jun 8;6(6):e20011. doi: 10.1371/journal.pone.0020011 (PMC3110596; doi:10.1371/journal.pone.0020011)

**Supporting Information – Appendix S5**

**A New Crocodile from the Upper Maastrichtian of Spain: Implications for the Initial Radiation of Crocodylids.**

**Eduardo Puértolas^1^, José I. Canudo^1^, Penélope Cruzado-Caballero^1^**

^1^Grupo Aragosaurus-IUCA (www.aragosaurus.com), Paleontología, Facultad de Ciencias, Universidad de Zaragoza, C/ Pedro Cerbuna 12, 50009 Zaragoza, España.

| **Measurements of the skull** | **mm** |
| --- | --- |
| 1. Skull, maximum length (from anterior end of premaxilla to quadrate condyle)^p^. | 196 |
| 2. Skull, medial length (from anterior end of premaxilla to parietal)^p^. | 164 |
| 3. Skull, maximum medial length (from anterior end of premaxilla to supraoccipital)^e^. | 175^e^ |
| 4. Skull, length of the skull table (along the dorsal midline)^p^. | 110^p^ |
| 5. Skull, width of the skull table (between posterior end of the squamosals). | 70 |
| 6. Skull, width of the skull table (between anterior margin of the supratemporal fenestrae). | 67 |
| 7. Skull, maximun width (between quadrate condyles)^p^. | 110^p^ |
| 8. Skull, minimum width (at the level of preserved premaxillary tooth)^p,e^. | 22^p^, 44^e^ |
| 9. Skull, width at the level of postorbital bar^p,e^. | 89^p^, 110^e^ |
| 10. Snout, maximun width (at the level of the anterior end of the orbits)^p,e^. | 52^p^, 100^e^ |
| 11. Snout, minimum width (at the level of the premaxillary-maxillary suture)^p,e^. | 22^p^, 44^e^ |
| 12. Snout, length (from anterior end of premaxilla to the anterior level of the orbits) | 107 |
| 13. Interorbital, minimum width (in the frontal). | 22 |
| 14. Frontal, width of the anterior process at the anterior margin of the orbits. | 9 |
| 15. Supratemporal fenestra, anteroposterior length. | 23 |
| 16. Supratemporal fenestra, lateromedial width. | 20 |
| 17. Infratemporal fenestra, anteroposterior length. | 27 |
| 18. Infratemporal fenestra, lateromedial width. | 15 |
| 19. Parietal, minimum interfenestral width. | 10 |
| 20. Frontal, total length. | 77 |
| 21. Naris, length. | 16 |
| 22. Naris, width ^p^. | 9^p^ |
| 23. Quadrate, average width. | 14 |
| 24. Pterygoid, width between the distal flank. | 50 |
| 25. Pterygoid, length in the lateral flank. | 40 |
| 26. Choana, length. | 10 |
| 27. Choana, width. | 12 |
| 28. Orbit, lenght. | 31 |
| 29. Orbit, width. | 25 |
| 30. Suborbital fenestra, anteroposterior length. | 65 |
| 31. Suborbital fenestra, maximum transverse width. | 27 |
| 32. Jugal, maximum length. | 80^p^ |
| 33. Orbital ramus of the Jugal, thick in the middle. | 17 |
| 34. Orbital ramus of the Jugal, length (up to postorbital bar). | 39 |
| 35. Jugal, infratemporal bar length (from postorbital bar)^p^. | 32 |
| 36. Jugal, average thickness of infratemporal bar. | 6 |
| 37. Jugal, average width of infratemporal bar. | 3 |
| 38. Postorbital bar, maximum anteroposterior diameter. | 14 |
| 39. Postorbital bar, minimum anteroposterior diameter. | 6 |
| 40. Foramen magnum, maximum transverse width^p^. | 12^p^ |
| 41. Foramen magnum, maximum dorsoventral thickness^p^. | 12^p^ |
| 42. Premaxilla, maximum width^p,e^. | 25^p^, 50^e^ |
| 43. Postsnout length to the supraoccipital^e^. | 68^e^ |
| 44. Postsnout length to the quadrate condyle. | 86 |
| 45. Width between the fifth maxillary teeth^p,e^. | 40^p^, 80^e^ |
| 46. Number of maxillary alveoli^p,e^. | 15^p^, 16-17^e^ |
| 47. Number of premaxillary alveoli^p,e^. | 2^p^, 4-5^e^ |
| **(Appendix S5: Dimensions of the holotype skull of *Arenysuchus*.** ^p^ preserved (bone not complete)**;** ^e^ estimated value (for reconstruction) | |

| **Ratios of the skull** | **%** |
| --- | --- |
| 1. Snout length/Medial Skull lenght^p^. | 107/164^p^ = **0.65** |
| 2. Snout length/Maximum medial Skull lenght^e^. | 107/175^e^ = **0.61** |
| 3. Snout, minimum width^p^/Snout, maximum width^p^. | 22^p^/52^p^ = **0.42** |
| 4. Snout, minimum width^e^/Snout, maximum width^e^. | 44^e^/100^e^ = **0.44** |
| 5. Width between the fifth premaxillary teeth^e^/Skull, maximum width^p^. | 80/110 = **0.72** |
| 6. Snout length/Postsnout length to the supraoccipital^e^. | 107/68^e^ = **1.57** |
| 7. Snout length/Postsnout length to the quadrate condyle. | 107/86 = **1.24** |
| 8. Snout, minimum width p/Premaxilla, maximum width^p^. | 22^p^/25^p^ = **0.88** |
| 9. Premaxilla, maximum width^p^/Skull, maximum width^p^. | 25^p^/110^p^ = **0.22** |
| 10. Naris, width^p^/Premaxilla, maximum width^p^. | 9^p^/25^p^ = **0.36** |
| 11. Supratemporal fenestra length /Orbit length. | 23/31 = **0.74** |
| 12. Infratemporal fenestra length /Supratemporal fenestra length. | 27/23 = **1.17** |
| 13. Parietal, minimum interfenestral width/Interorbital, minimum width. | 10/22 = **0.45** |
| 14. Frontal, width of the anterior process at the anterior margin of the orbits/Interorbital, minimum width. | 9/22 = **0.41** |
| 15. Width of the skull table (between the anterior margin of the supratemporal fenestra)/Skull, maximum width^p^. | 67/110^p^ = **0.61** |
| **(Appendix S5: Ratios of the holotype skull of *Arenysuchus*.** ^p^ preserved (bone not complete)**;** ^e^ estimated value (for reconstruction). **Calculations of proportions with** ^p^ preserved measures are perfectly valid in the bones preserved in one half with bilateral symmetry, since the entire skull would have a similar proportion. | |

**Appendix S5:** **Location of measurements (in mm) of the skull.** A, Dorsal; B, Lateral; C, Posterior; D, Ventral. The number in parentheses is the measurement of the first table.


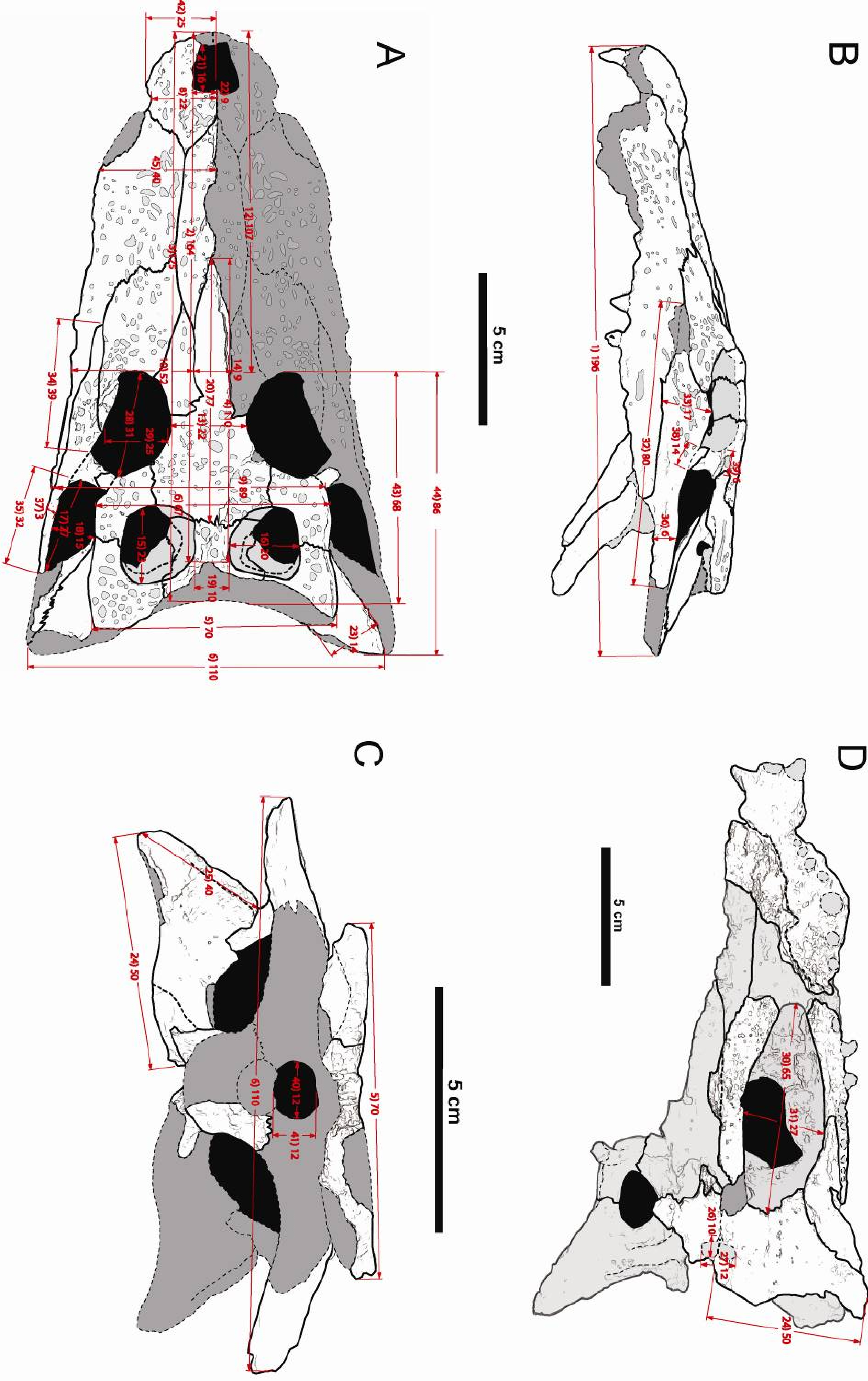

Supplement: Appendix S5 — Skull measurements. (DOC) [file pone.0020011.s005.doc]
